# Clinical characteristics and prognosis of 272 postterm choriocarcinoma patients at Peking Union Medical College Hospital: a retrospective cohort study

**DOI:** 10.1186/s12885-016-2383-1

**Published:** 2016-06-02

**Authors:** Jie Li, Junjun Yang, Pengfei Liu, Tong Ren, Jun Zhao, Fengzhi Feng, Xirun Wan, Yang Xiang

**Affiliations:** Departments of Obstetrics and Gynecology, Peking Union Medical College Hospital, Chinese Academy of Medical Sciences and Peking Union Medical College, No.1 Shuaifuyuan Wangfujing Dongcheng District, 100730 Beijing, People’s Republic of China

**Keywords:** Postterm choriocarcinoma, Prognosis factor, Survival, Combined chemotherapy

## Abstract

**Background:**

The objective of our study was to investigate the clinical characteristics and prognosis of postterm choriocarcinoma patients at Peking Union Medical College Hospital within the past 30 years.

**Methods:**

The clinical characteristics and pertinent follow-up data of 272 patients with postterm choriocarcinoma diagnosed from December 1985 through December 2014 in our hospital were reviewed. The clinical characteristics of two cohorts cut off at 2006 were compared using *χ*^2^ tests. Risk factors of prognosis were estimated by multivariate Cox proportional regression analysis.

**Results:**

The most common initial symptom was abnormal uterine bleeding. After individualized treatment 239 patients (87.9 %) achieved complete remission, including 140 patients received initial treatment of 5-fluorouracil-based multidrug chemotherapy. There were almost no statistically significant differences in the clinical characteristics and survival rates between the two cohorts. The results of the multivariate analysis showed that history of resistance to multidrug chemotherapy, liver metastasis and FIGO score greater than 12 were independent risk factors of prognosis.

**Conclusions:**

Postterm choriocarcinoma patients were usually accompanied by several high-risk factors that should received combined chemotherapy to prevent delay in adequate treatment. 5-fluorouracil-based multidrug chemotherapy, which has been applied at PUMCH for several decades, can be an effective initial treatment for postterm choriocarcinoma patients. More emphasis should be placed on those who have history of resistance to multidrug chemotherapy, liver metastasis or a FIGO score greater than 12.

**Electronic supplementary material:**

The online version of this article (doi:10.1186/s12885-016-2383-1) contains supplementary material, which is available to authorized users.

## Background

Gestational trophoblastic neoplasia (GTN) is considered to be one of the most curable human malignancies. Due to the precise measurement of human chorionic gonadotropin (hCG) levels, effective combination chemotherapy and other viable procedures such as surgeries, the cure rate can reach 90 % even for widespread metastases [1].

Choriocarcinoma, a type of gestational trophoblastic neoplasia, can follow any form of pregnancy, such as hydatidiform moles, abortion, term pregnancy or ectopic gestation [2]. Among these, the incidence of postterm choriocarcinoma has been reported to be as low as approximately 1 in 50,000 births [3]. Limited information is available about this specific form of choriocarcinoma due to its rarity. However several studies have identified that an antecedent term pregnancy could be an important risk factor of prognosis [4, 5]. Our previous study on cases between December 1985 to December 2006 also indicated that 108 patients (108/123 87.8 %) achieved complete remission (CR) and patients who had an interval between pregnancy and diagnosis greater than 12 months were more inclined toward an adverse prognosis than patients in other subgroups [6].

The objective of our present study was to investigate the clinical characteristics and prognosis of patients with postterm choriocarcinoma by surveying the cases treated at Peking Union Medical College Hospital (PUMCH) over the previous 30 years as well as compare the new data with our prior study to see if there were any differences in clinical characteristics. Furthermore, this paper gives a view of prognosis-related factors through the analysis of almost 270 cases for the first time.

## Methods

### Patients

From December 1985 through December 2014, 1418 patients with choriocarcinoma were admitted to PUMCH. Of these patients, 272 had an antecedent gestation of term pregnancy, including a new series of 149 cases from January 2007 through December 2014, which made the total prevalence rate 19.2 %. This study was approved by the ethics committee of Peking Union Medical College Hospital. The informed consent was waived because the study was retrospective in design and from public data-sets.

The age ranged from 21 to 57 years old (mean 31 years) with 24 (8.8 %) patients older than 40. 111 (40.8 %) patients in the cohort had an interval of more than 12 months from the index pregnancy. The International Federation of Gynecology and Obstetrics (FIGO) score ranged from 2 to 20 (mean 9). 87 (40.0 %) were low-risk patients and 185 were high-risk patients according to the new FIGO prognosis scoring system. Forty-six patients were diagnosed as stage I, 9 with stage II, 161 with stage III, and 56 with stage IV. The mean β-hCG levels before treatment in our hospital was 110,852 (range negative-3120000) mIU/ml. The percentage of patients with metastatic disease was 83.1 % (226/272). Seventy-seven patients with no less than two metastatic sites accounted for 34.1 % of the metastatic patients, which includes 22 cases of vaginal metastases, 6 ovary metastases, 77 lung metastases, 41 brain metastases, 10 liver metastases, 6 kidney metastases, 3 spleen metastases, 4 adrenal gland metastases, 4 gastrointestinal tract metastases and other distant metastases. Fifty-nine patients had two metastatic sites, 11 patients had three sites of metastases and the other 7 had four metastatic sites. One of the most common symptoms of postterm choriocarcinoma was abnormal uterine bleeding, which occurred in 202 (74.3 %) patients. The other symptoms included hemoptysis and central neural system symptoms (e.g., headache). The comparisons of clinical characteristics of the prior and new case series are displayed in Table 1.

**Table 1:** Comparisons of clinical characteristics

	1985–2006	2007–2014	*P* value
	*N* = 123	*N* = 149	
Mean age	30(22–57)	31(21–54)	0.25
Median gravidity	2(1–10)	2(1–8)	0.17
Median parity	1(1–9)	1(1–4)	0.56
Previous hydatidiform mole or invasive mole	21(17 %)	27(18 %)	0.82
Median interval from index pregnancy, months	7(0–216)	13(0–348)	0.002
Interval from index pregnancy <4 mo	47(38 %)	31(21 %)	0.002
Interval from index pregnancy >12 mo	36(29 %)	75(50 %)	0.000
Mean pretreatment β-hCG mIU/ml	119692 (6.5-3120000)	103554 (negative-2876010)	0.67
Pretreatment >400,000 β-hCG mIU/ml	5(4 %)	10(7 %)	0.34
Metastatic disease	98(80 %)	128(86 %)	0.17
Metastatic sites ≥2	38(31 %)	39(26 %)	0.39
Symptoms			
Abnormal uterine bleeding	91(74 %)	111(74 %)	0.92
Hemoptysis	14(11 %)	13(9 %)	0.47
Central neural system symptoms	12(10 %)	4(3 %)	0.17
Fetal death or stillbirth	3(2 %)	11(7 %)	0.07
Resistance to multiagent chemotherapy	49(40 %)	72(48 %)	0.16
Mean chemotherapy courses	8.5(1–27)	7.5(1–26)	0.44
Numbers of surgeries	75(61 %)	88(59 %)	0.75
Mean FIGO score	9.2(2–19)	9.4(2–22)	0.59
FIGO score >12	25(20 %)	31(21 %)	0.92
FIGO stage			
I	24(20 %)	22(15 %)	0.30
II	9(7 %)	0(0 %)	n/a^a^
III	66(54 %)	95(64 %)	0.09
IV	24(19 %)	32(21 %)	0.69

^a^ chi-square test undefined

### Treatment

All of the patients underwent a systematic evaluation including a review of medical history; physical examination; testing of complete blood count, serum biochemistry, liver and renal function, and serum β-hCG levels; and scanning by chest computed tomography (CT), pelvic and abdominal ultrasound or magnetic resonance imaging (MRI) and either brain CT or MRI when patients complained of neurological symptoms before receiving treatment in our hospital.

The standard treatments for patients with postterm choriocarcinoma were chemotherapy and sometimes surgery when the lesions appeared to be localized and resistant to chemotherapy. The chemotherapy agents used for the new and old cohort were similar. Chemotherapies utilizing 5-fluorouracil such as FAEV (5-fluorouracil/FUDR, actinomycin-D, vincristine, etoposide) and FAV (5-fluorouracil/FUDR, actinomycin-D, vincristine) were used as the first-line treatment in our hospital for untreated patients and patients who showed resistance to previously applied protocols that didn’t included 5-fluorouracil/FUDR. While the others transferred from other hospitals received multiple regimens as their first line chemotherapy agents. The details of FAEV or FAV protocol were as follows: vincristine 2 mg administered by bolus intravenously 3 h before actinomycin-D on day 1; actinomycin-D 200 μg/m^2^ administered by infusion more than 30 min daily on days 1–5; FUDR 800 mg/m^2^ administered by infusion more than 8 h daily on days 1–5; with or without etoposide 100 mg/m^2^ administered by infusion more than 30 min daily on days 1–5. The protocol was administered every 21 days. Patients discontinued the treatment if they had two or three additional courses of consolidation chemotherapy. The salvage chemotherapies included EMA/CO (etoposide, methotrexate, actinomycin-D/cyclophosphamide, vincristine), EMA/EP (etoposide, methotrexate, actinomycin-D/etoposide, cisplatin), TE/TP (paclitaxel, etoposide/paclitaxel, cisplatin) and other cisplatin-based chemotherapies. The toxicity of chemotherapy was evaluated for every cycle according to the WHO criteria. The hematologic toxicity, gastrointestinal reaction and alopecia were the most common side effects of the FAV/FAEV protocols. The occurrence rate of grade III ~ IV side effects, as much as 19.7 % (426/2163), were comparable to other multi-agents chemotherapies [1].

The mean courses of chemotherapy was 8 (1–27). One hundred twenty-one patients developed resistance to multiagent chemotherapies before they were admitted to our hospital, and 33 of these had previously received an EMA/CO regimen. The other 151 patients received either primary FAV or FAEV combination chemotherapy in our hospital. Within the whole cohort, 141 patients underwent 163 surgeries, including hysterectomy with or without bilateral salpingectomy and other surgeries to remove the chemoresistant metastatic lesions or in case of emergency. Forty-one patients with brain metastases received systematic chemotherapy and intrathecal injection of methotrexate (MTX).

### Efficacy assessment

Complete remission (CR) was defined as consecutively normal serum β-hCG levels for at least 4 weeks. Partial remission (PR) refers to conditions in which either lesions were reduced by 50 % or the serum β-hCG titers decreased by more than 50 %. Progression of disease (PD) was diagnosed when there were either consistent or rising serum β-hCG levels or new metastases. When β-hCG titers did not decline logarithmically or new metastases appeared after two or three cycles of chemotherapy, chemoresistance was diagnosed, and the patient was switched to salvage regimens.

### Statistical analysis

Results were presented as either the mean (SD) or median (range) and analyzed with the Statistical Package for the Social Science (SPSS 17.0, Armonk, NY, USA). The comparison of the mean between the case series was performed using t-tests. Ratios were compared using the chi-square test. The survival time was defined as the period between diagnosis and last follow-up or death. Loss of follow-up was considered to be censored data. The overall survival was analyzed by a Kaplan-Meier curve. Univariate analysis of the log rank test was used to estimate the influence of single factor on the overall survival, whereas the multivariate Cox proportional regression analysis was performed to determine the combination of factors that were statistically significant in the univariate analysis. The level of statistical significance was defined as *P* < 0.05.

## Results

### Prognosis

After systematic chemotherapies and certain individualized treatments, 239 (87.9 %) of the patients achieved CR, 8 achieved PR and the remaining 25 showed disease progression. Of the 151 patients who received either primary FAV or FAEV combination chemotherapy in our hospital, 140 (92.7 %) patients achieved CR. The CR rate of other patients who had a history of failed multidrug chemotherapy was only 81.8 % (99/121). The comparison of CR rate of our 272 post-term choriocarcinoma patients with CR rate of other patients treated for all gestational trophoblastic neoplasia at PUMCH for stage is shown in Table 2.

**Table 2:** Comparison of CR rate of 272 post-term choriocarcinoma with CR rate of other gestational trophoblastic neoplasia at PUMCH for stage

	Postterm choriocarcinoma		Other gestational trophoblstic neoplasia		*P* value
	No. of CR	CR rate	No. of CR	CR rate	
FIGO stage	239	87.9 %	2162	92.5 %	0.008
I	45	97.8 %	818	99.5 %	0.141
II	9	100 %	125	93.3 %	n/a^a^
III	145	90.1 %	1095	89.4 %	0.793
IV	40	71.4 %	124	79.0 %	0.249

^a^ chi-square test undefined

**Fig. 1:**
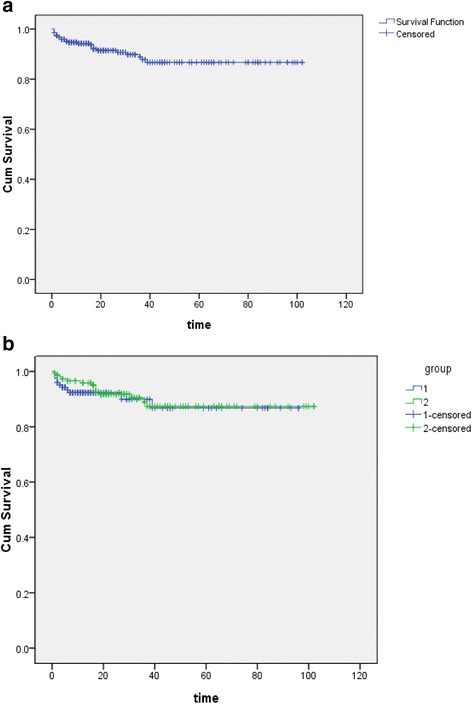
Kaplan-Meier curve for overall survival of **a** 272 postterm choriocarcinoma patients; **b** prior and new cohort respectively

Twenty-two patients died after receiving the initial treatment, 11 of whom (50 %) died of multiple organ failure due to the spread of the disease. Five of the patients had a cerebral hernia, and two had heart failure or renal failure due to heart and kidney metastases, respectively. 6 patients died of infectious shock, pneumorrhagia and respiratory failure caused by severe bone marrow suppression after chemotherapy. All of the surviving patients underwent follow-up either at outpatient clinics or through telephone interviews, except for 13 cases that were lost to follow up; the median follow-up time was 21 months (range 5–102 months). Seventeen cases relapsed within 3–24 months after initial remission, which included 3 cases of two-time relapses and one patient who relapsed three times. Fifteen of the relapses occurred within 6 months of remission and 3 patients died of disease progression. In total, 25 patients died of the disease, which made the overall 5-year survival rate 86.7 % (Fig. 1a).

### Comparisons of prior and new cohort

There were no statistically significant differences in the majority of the clinical characteristics between the two cohorts as displayed in Table 1. But the interval between the antecedent pregnancy and diagnosis appeared to be longer in the new cohort, extending from less than 1 month to 29 years (median 13 months) in the new cohort compared to the prior (*P* = 0.002, older series median interval 7 months). Nearly half of the patients in the new cohort had an interval of more than 12 months from the index pregnancy (*P* < 0.01). There was no significant difference between the prior and new cohorts with regard to the CR rate and survival rate (87.8 % vs. 87.9 %, 86.8 % vs. 87.3 %, respectively; *P* = 0.98, *P* = 0.61) either (Fig. 1b).

### Prognosis-related risk factors

The univariate analysis of prognosis-related factors on postterm choriocarcinoma patients is illustrated in Table 3. The interval from index pregnancy, the number of metastatic sites, a history of resistance to multiagent chemotherapy, liver metastasis and the FIGO score were related to the prognosis. All of the aforementioned factors were brought into the multivariate analysis. According to the results in Table 4, a history of resistance to multiagent chemotherapy [Relative Risk (RR) 2.613, 95 % CI 1.074–6.358, *P* = 0.034], liver metastasis (RR 5.393, 95 % CI 1.932–15.06, *P* = 0.001) and a FIGO score greater than 12 (RR 3.691, 95 % CI 1.660–8.205, *P* = 0.001) were independent risk factors for patient prognosis.

**Table 3:** The univariate analysis of prognosis related factors on 272 postterm choriocarcinoma patients

Clinical and demographics characteristics	5-year survival rate(%)	*P* value
Age		
<40 (*n* = 250)	88.0	
≥40 (*n* = 22)	72.7	0.13
Medical history of molar gestation		
Yes (*n* = 48)	84.3	
No (*n* = 224)	87.1	0.70
Interval from index pregnancy,month		
≤12 (*n* = 161)	93.2	
>12 (*n* = 111)	79.0	0.01
Pretreatment β-hCG,mIU/ml		
<100,000 (*n* = 220)	84.9	
≥ 100,000 (*n* = 52)	94.0	0.34
<400,000 (*n* = 257)	86.3	
≥ 400,000 (*n* = 15)	93.3	0.79
Metastatic sites		
1 (*n* = 195)	91.0	
≥2 (*n* = 77)	75.9	0.004
Abnormal uterine bleeding only		
Yes (*n* = 139)	88.4	
No (*n* = 133)	82.8	0.344
Fetal death or stillbirth		
Yes (*n* = 14)	92.9	
No (*n* = 258)	86.2	0.66
Resistance to multiagent chemotherapy		
Yes (*n* = 121)	76.3	
No (*n* = 151)	95.3	0.004
Brain metastasis		
Yes (*n* = 41)	80.6	
No (*n* = 231)	87.9	0.13
Kidney metastasis		
Yes (*n* = 6)	83.3	
No (*n* = 266)	86.7	0.55
Liver metastasis		
Yes (*n* = 10)	33.3	
No (*n* = 262)	89.0	0.000
Surgeries		
Yes (*n* = 141)	85.5	
No (*n* = 131)	87.4	0.25
FIGO score		
≤12 (*n* = 216)	90.9	
>12 (*n* = 56)	71.3	0.000

**Table 4:** The multivariate analysis of prognosis related factors on 272 postterm choriocarcinoma patients

Clinical and demographics characteristics	Relative risk	95 % CI	*P* value
Interval from index pregnancy >12 months	1.736	0.724–4.166	0.217
Metastatic sites ≥2	1.577	0.633–3.932	0.328
Resistance to multiagent chemotherapy	2.613	1.074–6.358	0.034
Liver metastasis	5.393	1.932–15.06	0.001
FIGO score >12	3.691	1.660–8.205	0.001

## Discussion

Postterm choriocarcinoma had seldom been reported because of its low incidence. Publications available are mostly case reports and occasionally case series of dozens of patients [6–10]. We conducted a retrospective study of 272 patients with regard to the risk factors that might be related to patient prognosis and compared a new cohort of 149 cases with that of a previously published series. Based on our observations, we concluded that the clinical characteristics of postterm choriocarcinoma in recent years had no obvious changes except for a later interval to diagnosis and delayed transferring to our hospital. The complete remission rate and 5-year overall survival rate of postterm choriocarcinoma patients in our cohorts were 87.9 and 86.7 %, respectively. A history of resistance to multiagent chemotherapy, liver metastasis and a FIGO score greater than 12 were shown to be independent risk factors of prognosis. To the best of our knowledge, we consider the sample size of this research to be the largest studied, and this is the first instance of applying multivariate analysis of risk factors for prognosis in postterm choriocarcinoma patients.

Most of the clinical characteristics of patients in our cohort were similar to other studies [7–10]. The most common symptom reported is abnormal uterine bleeding. Ryu et al. separated 24 patients into two groups according to their interval from the index pregnancy. The results showed that regardless of the time between the prior and current gestations, the most common initial symptom of postterm choriocarcinoma is irregular vaginal bleeding [8]. Ryu et al. also observed that persistent vaginal bleeding during pregnancy could be a sign of disease and required careful pathologic examination of the placenta to acquire an early diagnosis. Odunsi et al. reported a case of one patient who had secondary postpartum hemorrhaging and an anemic neonate, which was diagnosed as choriocarcinoma [11]. We considered a routine β -hCG test valuable for patients with abnormal vaginal bleeding either postpartum or during the pregnancy to exclude choriocarcinoma.

The median interval from the index pregnancy to time of diagnosis was 13 months in our new cohort. There was a statistically significant increase in the interval from index pregnancy (*P* = 0.002), as has also been previously reported [10]; however, the prognosis was unchanged. The longer intervals were perhaps due to delayed referrals to our hospital, which is considered to be a referral center for gestational trophoblastic disease that usually admits chemoresistant cases. Diver et al. observed more patients with fewer symptoms in a new series of patients who were diagnosed with choriocarcinoma, which could result in a delayed diagnosis [10]. Their study indicated that a timely diagnosis in postterm choriocarcinoma patients was required.

Almost all of the untreated patients in the new cohort received combined chemotherapies as their initial treatment. We conducted a comparison of CR rate of postterm choriocarcinoma patients with other patients treated for gestational trophoblastic neoplasia at the PUMCH for stage (Table 2). The results showed that there were no statistically significant difference when the CR rate was compared according to the stage subgroup. What’s more, the percentage of stage IV patients in postterm choriocarcinoma was higher than that in other patients treated for gestational trophoblastic neoplasia (20.7 % vs 6.7 %, *P* < 0.001). It appeared that stage might be a confounder of the association between postterm choriocarcinoma and relatively adverse prognosis. In other words, postterm choriocarcinoma was probably not a high-risk factor itself. These patients were possibly at higher risk because of other associated factors such as anatomical stage. Nevertheless, Lok et al. reported that choriocarcinoma following a term pregnancy was considered to be an independent high risk factor in the Netherlands because the high-risk status of postterm choriocarcinoma couldn’t be totally revealed by one or two traditional high-risk factors. Meanwhile, no subgroup could be identified in which single-agent chemotherapy would be used as initial treatment safely [7]. Lybol et al. conducted a retrospective study of the fatal cases of gestational trophoblastic neoplasia and found that 19 of 26 (73.1 %) cases were following a term pregnancy, and most of these deaths were due to a metastatic tumor. They suggested that gestational trophoblastic neoplasia following term pregnancy should be considered a high-risk disease, which probably contributed to a decline in fatal cases [5]. In our opinion, term pregnancy accounted for two points in the FIGO prognosis scoring system regardless of other risk factors. Whether term pregnancy is a high-risk factor itself or because of other associated factors still need more researches. But what’s more important is that the patients of postterm choriocarcinoma were usually accompanied by several high-risk factors that should received combined chemotherapy to prevent delay in adequate treatment. Chemotherapies containing 5-fluorouracil such as FAEV or FAV have been used in our hospital for several decades [12–14]. Almost all of the untreated patients and patients who exhibited resistance to protocols that lacked 5-fluorouracil/FUDR received either FAEV or FAV as their initial treatment. The study in the United States [10] also showed that the patients who underwent single-agent chemotherapy at first required altered combination chemotherapy to achieve remission.

Though the previous gestation of term pregnancy was considered a risk factor for prognosis in choriocarcinoma patients, the recently reported complete remission rate was approximately 86 % worldwide [7, 10]. Our data showed that the CR rate of 272 cases was 87.9% and both FAEV and FAV were effective combined chemotherapies when treating postterm choriocarcinoma patients in China and could be an alternative option for the initial chemotherapy. Twenty-five patients died from the disease during the treatment and follow-up period; 18 (84.0 %) of these patients died from either metastatic disease and or treatment-related issues. The overall 5-year survival rate of postterm choriocarcinoma patients was first reported as 86.7 % in this study, and there have been no improvements in the prognosis over the past 10 years. Another report revealed the same trend and speculated that the prognosis using more recent data could be better because of the initial combined chemotherapies, including EMA/CO [10].

The prognosis analysis in our prior study showed that an interval from the index pregnancy to diagnosis longer than 12 months, pretreatment β-hCG levels greater than 100,000 IU/L, FIGO stage IV and high-risk disease were related to an adverse prognosis [6]. Other literature also illustrated that a FIGO score greater than 8, multiple metastases, higher FIGO stages and pretreatment β-hCG levels greater than 40,000 IU/L were risk factors for a poor prognosis [15]. The present study discovered that the interval from index pregnancy to time of diagnosis, the number of metastatic sites, a history of resistance to multiagent chemotherapy, liver metastasis and FIGO score were related to the prognosis. The relationship between presenting symptoms and prognosis shown in Table 3 illustrated that the abnormal uterine bleeding alone had no significant effect on prognosis. However, Olive et al. found that presenting symptomatology other than abnormal uterine bleeding significantly decreased response to treatment in patients with postterm choriocarcinoma [16]. The presenting symptom could be the indication of tumor burden which was usually revealed by the serum β-hCG levels. According to our results, β-hCG levels had no significant effect on prognosis, either. Whether the presenting symptom was a significant prognostic factor or not still remains a question. After multivariate analysis, a history of resistance to multiagent chemotherapy, liver metastasis and a FIGO score greater than 12 were independent risk factors of the prognosis. Although the number of metastatic sites was significant only in the univariate analysis, we still considered it an important factor related to patient prognosis. After analyzing the data of patients metastases ≥2 sites, we found that nearly 80 % (60/77) of them had the history of resistance to multiagent chemotherapy. The effect of metastases ≥ 2 sites on prognosis might be explained by the factor of resistance to multiagent chemotherapy. On the other hand, there were only a few patients with more than two metastatic sites. More cases with more than two metastatic sites are necessary to determine whether the number of metastatic sites (e.g., >2) is related to the prognosis. Kidney metastasis concomitant with other distant metastases was considered a risk factor in some reports [13, 14]. Because of the small sample size, the survival rate of patients with or without kidney metastasis had no statistically significant difference in our analysis (*P* = 0.55). The results of multivariate analysis were similar with a study conducted in FIGO stage IV patients [13].

Given the rarity of choriocarcinoma following term pregnancy, we can not conduct a prospective study because the selection bias of our research is difficult to avoid. However, this study, as the largest retrospective cohort study to date, provides invaluable data that may aid in better understanding this disease.

## Conclusions

Postterm choriocarcinoma patients were usually accompanied by several high-risk factors that should received combined chemotherapy to prevent delay in adequate treatment. 5-fluorouracil-based multidrug chemotherapy, which has been applied at PUMCH for several decades, can be an effective initial treatment for postterm choriocarcinoma patients. More emphasis should be placed on those who have history of resistance to multidrug chemotherapy, liver metastasis or a FIGO score greater than 12. The relationships between the prognosis and multiple metastases or kidney metastasis require additional study.

## Abbreviations

CR, complete remission; CT, computed tomography; EMA/CO, etoposide, methotrexate, actinomycin-D/cyclophosphamide, vincristine; EMA/EP, etoposide, methotrexate, actinomycin-D/etoposide, cisplatin; FAEV, 5-fluorouracil/FUDR, actinomycin-D, vincristine, etoposide; FAV, 5-fluorouracil/FUDR, actinomycin-D, vincristine; FIGO, International Federation of Gynecology and Obstetrics; GTN, gestational trophoblastic neoplasia; hCG, human chorionic gonadotropin; MRI, magnetic resonance imaging; PD, progression of disease; PR, partial remission; PUMCH, Peking Union Medical College Hospital; TE/TP, paclitaxel, etoposide/paclitaxel, cisplatin.

## Additional file

Additional file 1. The prognosis related factors of every patient were listed in the worksheet. (XLSX 27.7 kb)
